# Evidence for contribution of common genetic variants within chromosome 8p21.2-8p21.1 to restricted and repetitive behaviors in autism spectrum disorders

**DOI:** 10.1186/s12864-016-2475-y

**Published:** 2016-03-01

**Authors:** Yu Tao, Hui Gao, Benjamin Ackerman, Wei Guo, David Saffen, Yin Yao Shugart

**Affiliations:** Department of Cellular and Genetic Medicine, School of Basic Medical Sciences, Fudan University, 130Dong’an Road, Shanghai, 200032 China; JohnsHopkins University, Baltimore, MD USA; Unit on Statistical Genomics, Intramural Research Program, National Institute of Mental Health, National Institute of Health, Bethesda, MD USA

**Keywords:** Restricted and Repetitive Behaviors (RRB), Genome-Wide Association Study, Autism Spectrum Disorders (ASD), Autism Diagnostic Interview-Revised (ADI-R)

## Abstract

**Background:**

Restricted and Repetitive Behaviors (RRB), one of the core symptom categories for Autism Spectrum Disorders (ASD), comprises heterogeneous groups of behaviors. Previous research indicates that there are two or more factors (subcategories) within the RRB domain. In an effort to identify common variants associated with RRB, we have carried out a genome-wide association study (GWAS) using the Autism Genetic Resource Exchange (AGRE) dataset (*n* = 1,335, all ASD probands of European ancestry) for each identified RRB subcategory, while allowing for comparisons of associated single nucleotide polymorphisms (SNPs) with associated SNPs in the same set of probands analyzed using all the RRB subcategories as phenotypes in a multivariate linear mixed model. The top ranked SNPs were then explored in an independent dataset.

**Results:**

Using principal component analysis of item scores obtained from Autism Diagnostic Interview-Revised (ADI-R), two distinct subcategories within Restricted and Repetitive Behaviors were identified: Repetitive Sensory Motor (RSM) and Insistence on Sameness (IS). Quantitative RSM and IS scores were subsequently used as phenotypes in a GWAS using the AGRE ASD cohort. Although no associated SNPs with genome-wide significance (*P* < 5.0E-08) were detected when RSM or IS were analyzed independently, three SNPs approached genome-wide significance when RSM and IS were considered together using multivariate association analysis. These included the top IS-associated SNP, rs62503729 (*P*-value = 6.48E-08), which is located within chromosome 8p21.2-8p21.1, a locus previously linked to schizophrenia. Notably, all of the most significantly associated SNPs are located in close proximity to *STMN4* and *PTK2B*, genes previously shown to function in neuron development. In addition, several of the top-ranked SNPs showed correlations with *STMN4* mRNA expression in adult CEU (Caucasian and European descent) human prefrontal cortex. However, the association signals within chromosome 8p21.2-8p21.1 failed to replicate in an independent sample of 2,588 ASD probands; the insufficient sample size and between-study heterogeneity are possible explanations for the non-replication.

**Conclusions:**

Our analysis indicates that RRB in ASD can be represented by two distinct subcategories: RSM and IS. Subsequent univariate and multivariate genome-wide association studies of these RRB subcategories enabled the detection of associated SNPs at 8p21.2-8p21.1. Although these results did not replicate in an independent ASD dataset, genomic features of this region and pathway analysis suggest that common variants in 8p21.2-8p21.1 may contribute to RRB, particularly IS. Together, these observations warrant future studies to elucidate the possible contributions of common variants in 8p21.2-8p21.1 to the etiology of RSM and IS in ASD.

**Electronic supplementary material:**

The online version of this article (doi:10.1186/s12864-016-2475-y) contains supplementary material, which is available to authorized users.

## Background

Autism Spectrum Disorder (ASD) is characterized by impaired reciprocal social interactions, delayed or aberrant communication, and the presence of restricted and repetitive behaviors, frequently with restricted interests [1]. These disabilities often confer significant lifelong burdens on individuals with ASD. This fact, together with the high ASD prevalence in the general population, makes ASD a major challenge for public health systems. [2–5] Based on heritability estimates as high as 70- 90 % in twin and family studies [6], great effort has been devoted to elucidating the genetic mechanism of ASD. However, it has been difficult to identify any individual genetic factors that confer even moderate risk [7, 8].

Genome-wide association studies (GWAS) have implicated the region on chromosome 5p14.1 between *CDH9* and *CDH10* as the first potential common genetic risk factor for ASD in Caucasian populations [9, 10]. Replication in independent GWAS, however, has frequently not been achieved for many candidate loci for ASD [11–14]. Phenotype and genetic heterogeneity between patients are conjectured to greatly reduce the power of overall genome-wide case-control studies in ASD, and is a likely explanation for the lack of replication and much of the ‘missing heritability’ in this complex disease [15]. Various attempts have been made to reduce heterogeneity in large-scale genetic studies of ASD. One proposed approach to increase statistical power to detect pathogenic loci is to design genetic association studies focusing on ASD sub-phenotypes [16–19].

Restricted and repetitive behaviors (RRB) are a core symptom of ASD [20]. Previous studies have shown that RRB have an underlying genetic component and may be influenced by genes independent of those associated with the social or communication deficits [21–23]. Moreover, Autism Diagnostic Interview-Revised (ADI-R), a gold-standard diagnostic tool for ASD [24], provides widely-accepted quantitative measures for RRB [25, 26], making it a promising sub-phenotype for association studies. Since RRB comprises heterogeneous groups of behaviors [27, 28], research during the last decade has used factor-analysis to examine the structure of RRB using different subsets of ADI-R items and subpopulations of ASD individuals that differ in symptom severity and/or ethnicity [27, 29–32]. Remarkably, in spite of their methodological differences, many of these analyses converge on a two-factor solution for RRB comprising ‘repetitive sensory-motor’ (RSM) and ‘insistence on sameness’ (IS). The RSM subcategory quantifies motor mannerisms, sensory seeking behaviors, and the repetitive use of objects, whereas the IS subcategory quantifies compulsions, rituals and difficulties with changes in routine [30].

IS and RSM were found to be differentially related to other ASD variables. Specifically, high correlations were found between RSM, but not IS, with IQ, additional adaptive behaviors, and age at first words and phrases [29, 30, 33], suggesting that, compared to IS, RSM may be more correlated with ASD severity [33]. Studies also indicated that the IS subcategory might be under stronger additive genetic control than the RSM subcategory. Whereas significant familial aggregation of the IS subcategory has been consistently reported [31, 32], no significant concordance for familial aggregation has been reported for RSM [25, 27, 31, 34].

Behavioral subcategories that differ in behavioral correlates and familiality are of particular interest to researchers investigating the genetic components that underlie ASD sub-phenotypes. In a recent genome-wide linkage analysis [25], RSM and IS subcategories were linked to various chromosomal regions that only partially overlapped regions previously identified using ASD diagnosis as the phenotype.

In the current work, we explored the underlying structure of RRB using two independent, publicly available ASD datasets. In an effort to identify SNP markers, candidate genes and biological pathways associated with RRB, the empirically derived RRB subcategories were then used as quantitative traits for GWAS. The observation that both univariate and multivariate linear mixed models identified associated SNPs within 8p21.2-21.1 in the discovery dataset, provides the first evidence that genetic variation in this region influences RRB phenotypes in ASD. Further studies are needed to confirm the association signals within chromosome 8p21.2-8p21.1, since replication was not obtained in an independent sample of 2,588 ASD probands, possibly due to insufficient sample size and between-study heterogeneity.

## Methods

### Ethics statement

This study was approved by the ethics committee of the School of Basic Medical, Fudan University, China (IRB#2010CB529601). All the genetic data and phenotype data used is previously published and publicly available. Written informed consent was previously obtained from all individuals and procedures had approval from institutional review boards from all the institutions involved in recruitment and research, following national and international ethical and legal regulations and the principles of the Declaration of Helsinki.

### Dataset demographics

The discovery dataset comprised individuals in the Autism Genetic Resource Exchange family-based dataset (AGRE: http://www.agre.org) [35]. AGRE has obtained informed consent from all individuals listed in their pedigree catalogue. Individuals with ASD in the AGRE cohort were diagnosed using both the Autism Diagnostic Interview-Revised (ADI-R) [24] and Autism Diagnostic Observation Schedule (ADOS) [36], widely considered to be the gold-standard diagnostic instruments for ASD. Individuals with possible non-idiopathic ASD (e.g., patients with significant chromosomal abnormalities, premature birth, or comorbid disorders) were excluded. All subjects were genotyped using the Illumina HumanHap550 BeadChip. Genotyping details and other important information have been previously described [9]. A “cleaned” version of the raw AGRE genetic data, designated CHOP.clean100121, was downloaded from the AGRE website (4,327 subjects). Following the method described in the supplement section of the study by Wang et al. [9], population structure was examined based on the first two principal components obtained by multidimensional scaling (MDS) of a matrix of pairwise IBS (Identical By State) values between these individuals. Individuals of European ancestry were selected based on principal component 1 scores less than 0.0, and principal component 2 scores between -0.02 and 0.02. A total of 806 ASD families (3,455 individuals) were inferred to have European ancestry using the above procedure (Additional file 1). Because ADI-R record were available only for ASD probands rather than for all pedigree members, the final discovery dataset comprised 1,335 probands, ranging in age from 1.8 to 44 years (mean = 8.00 years, SD = 4.87), and were predominately male (78.7 %). The sample size of each computer-scored diagnostic group defined by AGRE was: Autism 1,152 (86.3 %), Not Quite Autism (NQA) 57 (4.27 %), and Broad Spectrum (BS) 126 (9.44 %). The specific criteria for these classifications are given on the AGRE website. Detailed information for all individuals can be found in Additional file 2.

The dataset used for replication comprised individuals in Simons Simplex Collection (SSC, version 15), a genetic study limited to families with one child with ASD (the proband). Previous reports have described the SSC data collection process, as well as the extensive phenotypic data available [37]. Informed consent was obtained at each data collection site included in the SSC. Our group obtained phenotype and genotype data for 2,591 ASD families, from which 2,588 probands with ADI-R records were selected for analysis, These probands ranged in age from 1 to 108 years (mean = 21.46 years, SD = 13.96), and were predominately male (86.67 %). The sample size of each diagnosed status defined by ADI-R was Autism: 2,346 (90.65 %), and Autism Spectrum Disorder (ASD): 242 (9.35 %). Detailed information for all the individuals can be found in Additional file 3.

### Diagnostic instruments

The Autism Diagnostic Interview-Revised (ADI-R) instrument is a standardized parent interview designed to assess the presence and severity of symptoms based on the DSM-IV criteria for ASD [24]. Items designed for interviews fall within three diagnostic categories: i) social, ii) communication and iii) restricted and repetitive behaviors. Two scores are given for most items: a ‘current’ score, which assesses behavior during the past 3 months, and an ‘ever’ score, which assesses behavior in early childhood or at its greatest severity. We used the ‘current’ score in each item to avoid potential retrospective bias that could result from using the ‘ever’ score. The full range of each item scores (0–3) was used to provide maximal information of severity. Scores of 7 (“definite abnormality in the general area of the coding, but not of the type specified”), 8 (“not applicable”), and 9 (“not known or not asked”) given under certain circumstances were all converted to 0, according to the algorithm listed in ADI-R [38].

### Genotype imputation

Imputation of genotypes for autosomal SNPs was performed using IMPUTE version 2.2.2 [39, 40]. The reference panel used was the 1000 Genomes Phase I integrated haplotypes, which were produced using SHAPEIT2 [41] and released in June 2014 (http://mathgen.stats.ox.ac.uk/impute/impute_v2.html#reference). Imputed SNPs with low imputation quality (R^2^ or info scores < 0.3) or minor allele frequencies (MAF) < 5 % were excluded.

### Statistical analysis

#### PCA analysis of RRB items

Factor analysis was carried out using Principal Components Analysis (PCA) with varimax rotation on 11 RRB items from the ADI-R using R [42, 43]. These items were previously included in the RRB subdomains, RSM and IS by S. L. Bishop and colleagues using exploratory factor analysis [27]. Similar to previous factor analyses of the ADI-R [30, 34], we employed a cutoff of 0.30 for the inclusion of an item in a factor. Correlation analyses were conducted to examine relationships between RRB subcategories.

#### Assessment of familiality of RRB subcategories

To investigate potential familial relationships in the empirically derived subcategories, intraclass correlations (ICCs) between sibling pairs with any ASD diagnosis (i.e., Autism, NQA, or BS) from the multiplex families were calculated (monozygotic twins were excluded) [30]. Affected sib pairs of each multiplex family were included in ICCs calculation (*N* = 200 from 100 families). This analysis was only done using the AGRE dataset, since SSC was limited to families with only one ASD child.

#### Genome-wide association analyses

The association of SNPs with the RRB subcategories was measured using a novel Genome-wide Efficient Mixed-Model Association (GEMMA) approach developed by Zhou and Stephens [44]. Briefly, GEMMA fits univariate linear mixed models for associations with single phenotypes or multivariate linear mixed models for simultaneous associations with multiple phenotypes, while controlling for sample relatedness and potential population stratification. (For addition details see [44–46]). GEMMA was downloaded from http://www.xzlab.org/software.html. Raw item scores from ADI-R score sheets of ASD probands were summed for each subcategory of RRB identified by PCA analysis. Sex and age at ADI-R standardized residuals of the summed scores of each subcategory were calculated using multivariate regression. These residuals were normalized following Tukey’s formula using SPSS and then used as phenotypes in genome-wide association analysis. Autosomal chromosome association results were retained [47]. First, associations between SNPs and each subcategory were tested using the GEMMA program based on a univariate linear mixed model, while applying a correction for sample structure (population stratification and hidden relatedness) through a pairwise relatedness matrix derived from SNP genotypes. Second, the GEMMA program was used to investigate associations between SNPs and RRB sub-categories in a multivariate analyses model to estimate the robustness of the associations. The use of multivariate methods has been recommended, because multivariate analyses may increase power not only to detect genetic variants that affect only one of the multiple correlated phenotypes, but also pleiotropic genetic variants [44–46].

Following association analysis, statistical evidence for association was evaluated by carrying out genome-wide association analysis for 1000 permutations of phenotypes. To ease the computational burden, these analyses were performed for genotyped SNPs only. To estimate genomic inflation factors for all the distributions of *P*-values, lambda genomic control (lambdaGC) values were calculated as the ratio of the median of the empirically observed distributions of the test statistic to the expected median. The empirical *p*-value for the original association was calculated as the proportion of permutation replicates with lambdaGC values greater than the lambdaGC value of the original distribution.

### *Cis*-eQTL analysis for the candidate loci

Expression quantitative trait loci (eQTL) analysis was conducted using gene expression data obtained using adult human prefrontal cortex obtained from the “BrainCloud” study (http://braincloud.jhmi.edu) [48]. Only individuals of European ancestry (*n* = 112) were included in the analysis. Individual-level mRNA expression data were downloaded from the Gene Expression Omnibus (accession number GSE30272) and genotype data were obtained from dbGaP (accession number phs000417.v1.p10.) Linear regression models were used to identify SNPs located within 500 kb of the transcript being tested (*cis*-eQTL) with statistically significant correlations between genotype and mRNA expression levels, with RNA integrity numbers (RIN) and age included as covariates. Genotype imputation for chromosome regions of interest was performed as described elsewhere [49].

### Functional enrichment analysis

To identify plausible pathways associated with RRB, we expanded our focus beyond single variants by performing functional enrichment analysis using the web-accessible bioinformatics tool, Database for Annotation, Visualization and Integrated Discovery (DAVID) [50, 51]. Since DAVID can only handle gene lists, SNPs with an association P-value smaller than 0.01 were used to compile a list of genes for further analysis (i.e., a list of all genes that contain associated SNPs or are the nearest genes to associated intergenic SNPs). Analysis was performed using the software’s Functional Annotation Clustering option. The“Functional Annotation Clustering” tool identifies gene annotation terms that are enriched in the input gene list compared to the gene list from entire human genome and ranks the terms according to their enrichment *P*-values calculated using Fisher’s exact test. Subsequently, “clusters”of related gene annotation terms that are enriched in the input gene list are assigned an “enrichment score” (ES), defined as the geometric mean of the log_10_-transformed *P*-values for all gene annotation terms in the cluster. Enrichment scores > 1.3 are considered to be nominally significant.

## Results

### Discovery (AGRE) dataset

#### Factor substructure of RRB

Descriptive statistics of 11 ADI-R items used in these analyses are listed in Table 1. Detailed information regarding each item is given in Additional file 4. The two-factor solution provided a satisfactory fit to 11 ADI-R items in the PCA analysis (Chi Square [df = 34] = 128.61, *P*-value = 6.38E-13). Using a cutoff of ≥ 0.3 for the inclusion of items in a respective factor, 9 out of 11 items loaded on the two factors (Table 2). Four items loaded on Factor 1 and five items loaded on Factor 2. Loadings on Factor 1 (Repetitive Sensory Motor: RSM subcategory) ranged from 0.46 to 0.75. Loadings on Factor 2 (Insistence on Sameness: IS subcategory) ranged from 0.30 to 0.68.Sum scores for RSM and IS were calculated by summing the scores of items included in each factor. RSM and IS gave similar score distributions that spanned the full range of possible scores (i.e., 0–11 for RSM, 0–15 for IS). For the entire sample, the mean RSM score was 4.096 (SD = 2.82), and the mean IS score was 3.73 (SD = 2.92) (Additional file 5). Together, the two subcategories accounted for 42 % of the variance of RRB. RSM and IS subcategory scores were correlated at *r* = 0.204 (*P*-value < 0.001), indicating that they share 4 % of their variance (r^2^ = 0.04).

**Table 1:** Descriptive statistics of 11 “current score” items from ADI-R in the AGRE cohort

ADI-R items from RRB domain	*N*	Mean	Std. Deviation	Range
Circumscribed interests	1335	0.778	1.068	0-3
Difficulties with change	1335	0.885	0.979	0-3
Resistance to change	1335	0.331	0.702	0-3
Compulsions/rituals	1335	0.738	1.025	0-3
Sensitivity to noise	1335	0.993	1.015	0-3
Abnormal/Idiosyncratic response	1335	0.996	0.975	0-3
Unusual preoccupations	1335	0.396	0.803	0-3
Repetitive use of objects	1335	1.227	1.121	0-3
Unusual sensory interests	1335	1.038	0.801	0-2
Hand and finger mannerisms	1335	0.905	0.967	0-3
Complex mannerisms or stereotyped body movements	1335	0.925	1.004	0-3

**Table 2:** Two-Factor solution for the Restricted and Repetitive Behaviors (RRB) in the AGRE cohort using PCA with varimax rotation

ADI-R items from RRB domain	Factor1	Factor2
	Repetitive Sensory Motor (RSM)	Insistence On Sameness (IS)
Unusual sensory interests	**0.75**	0.08
Repetitive use of objects	**0.71**	0.07
Hand and finger mannerisms	**0.48**	0.1
Complex mannerisms or stereotyped body movements	**0.46**	0.12
Difficulties with change	0.03	**0.68**
Resistance to change	0.13	**0.53**
Circumscribed interests	-0.11	**0.30**
Compulsions/rituals	0.16	**0.49**
Abnormal/Idiosyncratic response	0.25	**0.36**
Unusual preoccupations	0.12	0.22
Sensitivity to noise	0.1	0.29

Note: Factor loadings of those items which exceed 0.30 are bolded

**Fig. 1:**
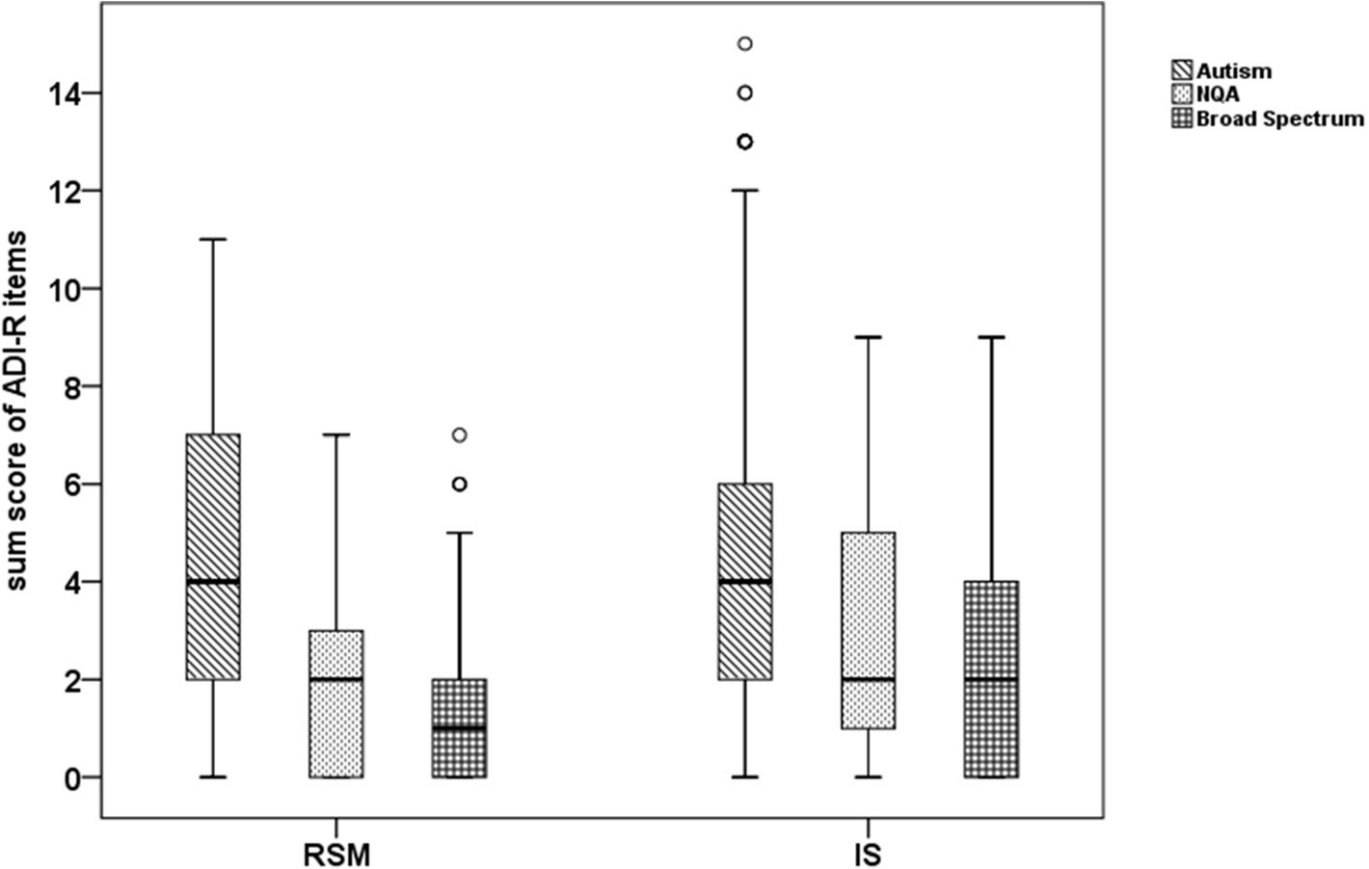
Box plots of RSM and IS scores for ASD probands in the AGRE cohort. The y-axis indicates sum scores for RSM and IS, calculated by summing the scores of items included in each RRB subcategory. Scores for each RRB subcategory were calcluated separately for indivuduals within the three status categories defined by AGRE: Autism, Not Quite Autism (NQA) and Broad Spectrum

ANOVA was conducted to compare mean scores for each derived subcategory with respect to status category defined by AGRE: Autism, NQA (Not Quite Autism), and Broad Spectrum [52]. Both for RSM and IS, significant differences were detected among the three status categories (F = 101.8, *P*-value < 0.0001; F = 28.4, *P*-value < 0.0001). The Tukey HSD post-hoc test indicated that both RSM and IS scores for individuals in the Autism category were significantly higher than for individuals in the NQA or BS categories, who did not differ in their scores (Fig. 1).

#### Familiality of RRB

To test potential familial relationships between sibling pairs in our data, we calculated interclass correlations (ICCs) between sibling pairs for RSM and IS and the original RRB domain from ADI-R. A significant family genetic effect was shown for IS (ICC = 0.405, *P*-value = 0.005), while the ICC for RSM was 0.042 (*P*-value =0.416) and the ICC for RRB was 0.359 (*P*-value = 0.015).

#### Genome-wide association analysis

**Fig. 2:**
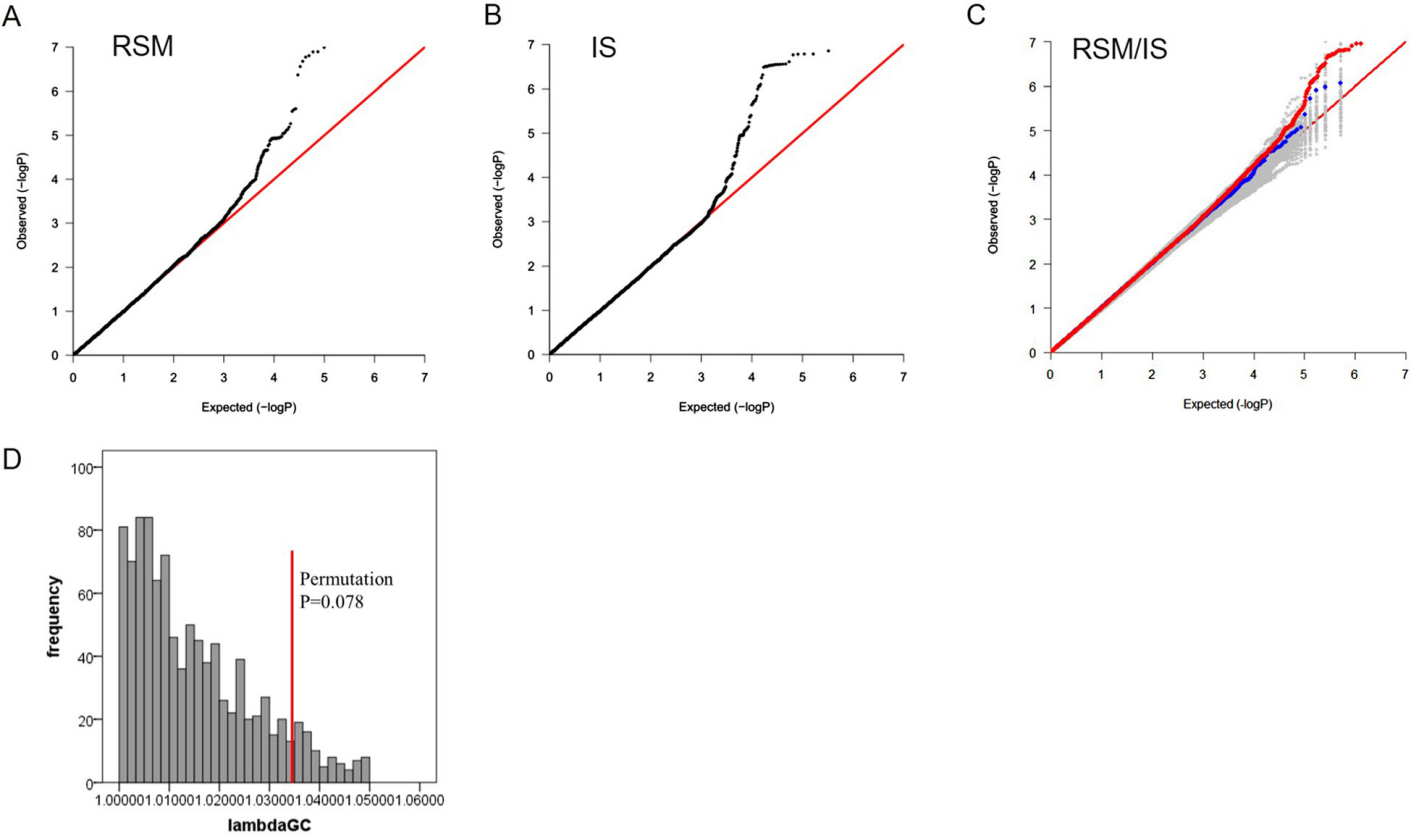
Quantile-Quantile plots of *P*-values for association with IS, RSM or RSM/IS in the AGRE GWAS cohort. Q-Q plots compare log_10_-transformed observed P-values [−log_10_(Observed P)] to log_10_-transformed *P*-values expected under the null hypothesis of no association [−log_10_(Expected P)]. **a** and **b** Q–Q plots obtained for association of GWAS SNPs with RSM or IS based on a univariate linear mixed model. **c** For associations of GWAS SNPs with RSM/IS based on a multivariate linear mixed model, QQ-plots of the true association statistics for genotyped and imputed SNPs are shown in red, genotyped SNPs only in blue, 1000 phenotype-permutated replicates in gray. **d** The true lambdaGC value (1.0355) is shown as a red vertical line and the distributions of lambdaGC values from 1000 phenotype-permutated replicates is shown as a histogram

**Fig. 3:**
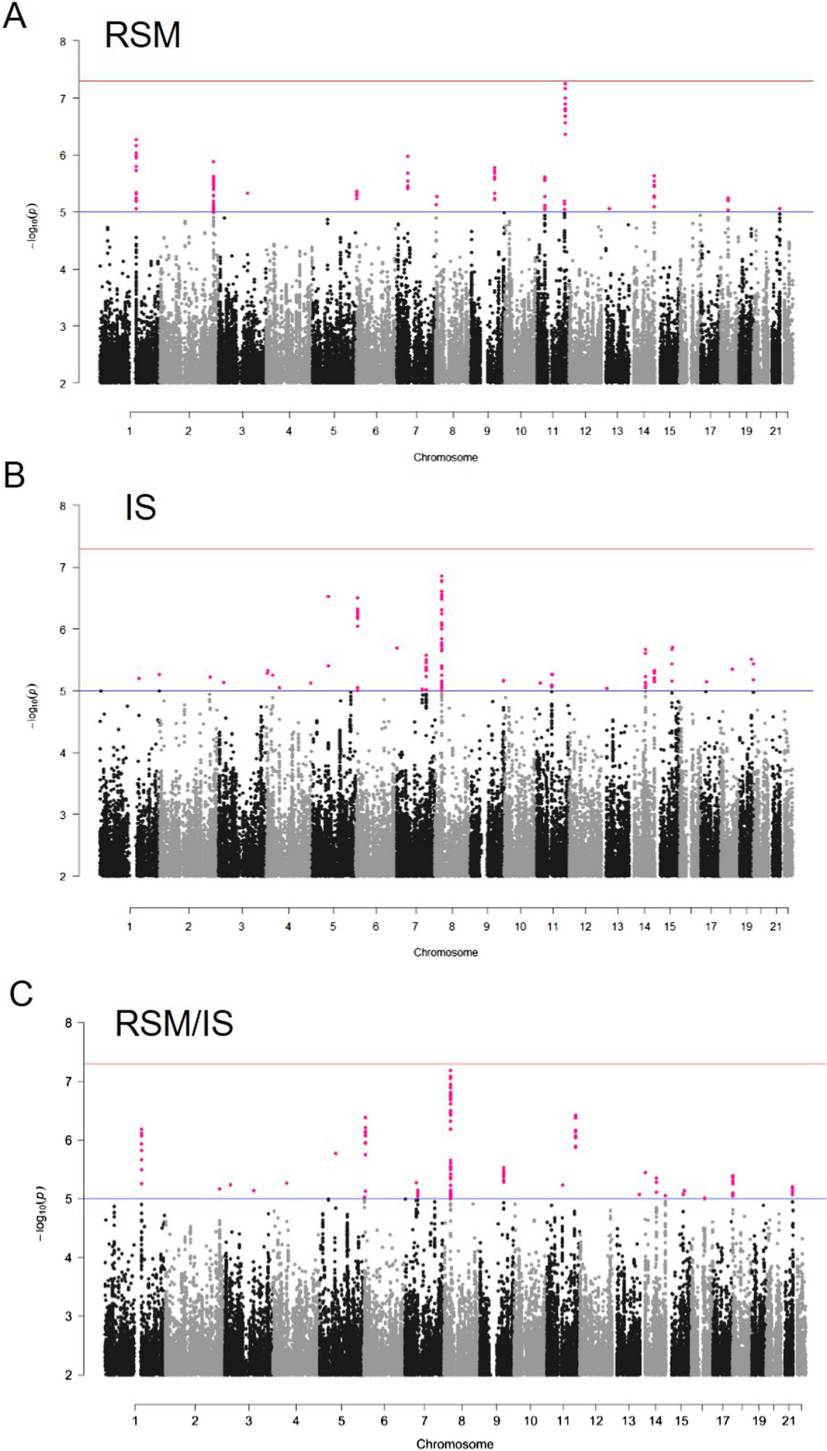
Manhattan plots for RSM (**a**), IS (**b**) and RSM/IS (**c**) for the AGRE cohort. The x-axis indicates the position of each SNP on individual autosomal chromosomes and the y-axis indicates –log_10_ transformed GEMMA P-values. Red line: genome-wide significance threshold (*P* = 5.0E-08). Blue line: threshold for “suggestive significance” (*P* = 5.0E-05)

After filtering for minor allele frequency (MAF) < 5 % and info score r^2^ < 0.3, 6,066,362 genotyped or imputed SNPs were tested for association with standardized and normalized RSM (univariate linear mixed model), IS (univariate linear mixed model), and RSM/IS (multivariate linear mixed model) scores using GEMMA mixed model association analyses. Quantile-Quantile (QQ) plots of *P*-values for association with IS or RSM in the AGRE cohort GWAS are shown in Fig. 2. QQ plots of *P*-values for association with RSM/IS and permuted RSM/IS phenotypes are also shown, with the true distribution compared to the permuted distributions (Fig. 2c). The true lambdaGC value was 1.0355, while the permutation *P*-value for RSM/IS association result was 0.078 based on the distribution of lamdaGC values obtain from all permutations analyzed (Fig. 2d). These results suggest little evidence for inflation of *P*-values due to stratification or other confounding biases, but provide compelling evidence for RRB association. The Manhattan plots for associations of SNPs with RSM、IS or RSM/IS quantified using GEMMA are shown in Fig. 3.

We failed to detect genome-wide significant associations (*P*-value < 5.0E-8) with RSM or IS in the AGRE cohort. However, rs11512467 at 11q23 showed an association signal close to genome-wide significance for RSM (5.67E-08) and ten SNPs at this locus had *P*-values below 4.27E-07, all located within 20 kb upstream from *IL10RA* (Fig. 3a), Table 3). The strongest univariate association signals for IS were detected at 8p21.2-8p21.1, including the top 4 significant SNPs/rs62503729 (*P*-value = 1.39E-07), rs13278976 (*P*-value = 1.64E-07), rs13270725 (*P*-value = 1.64E-07) and rs35240189 (*P*-value = 1.65E-07), located within 10 kb downstream of the *STMN4* gene (Fig. 3b), Table 4). Several other regions showing suggestive association signals (*P*-value < E-06) for RSM or IS were also detected, such as 1q21-1q21.2 (within the 3’-UTR of *MCL1* (Myeloid cell leukemia 1) and an intron on *ENSA* (endosulfine alpha)), 6p24.3 (in an intron of *TXNDC5* (thioredoxin domain containing 5)) (Table 3, Table 4). These regions cannot be easily implicated in RRB, but may still provide new insights, if confirmed in follow-up studies with larger samples or sequencing validations.

**Table 3:** List of the most significant associations with RSM (*P*-value < E-06) in the AGRE cohort

CHR	SNP	BP (hg19)	Effect allele	F^a^	Nearest Gene	beta	se	P(Wald)	Type
11	rs11512467	117835174	C	0.577	*IL10RA* (20 kb upstream)	0.5896	0.1080	5.67E-08	Imputed
11	rs12790242	117842292	G	0.579	*IL10RA* (20 kb upstream)	0.5930	0.1093	6.86E-08	Imputed
11	rs11216657	117839187	A	0.573	*IL10RA* (20 kb upstream)	0.5819	0.1087	1.01E-07	Imputed
11	rs73026358	117838810	C	0.574	*IL10RA* (20 kb upstream)	0.5777	0.1088	1.27E-07	Imputed
11	rs11216656	117838400	G	0.584	*IL10RA* (20 kb upstream)	0.5778	0.1088	1.28E-07	Imputed
11	rs2512158	117823425	G	0.576	*IL10RA* (20 kb upstream)	0.5693	0.1080	1.56E-07	Genotyped
11	rs7122174	117842533	A	0.43	*IL10RA* (20 kb upstream)	0.5772	0.1097	1.68E-07	Imputed
11	rs4938465	117844785	T	0.45	*IL10RA* (20 kb upstream)	0.5826	0.1116	2.09E-07	Imputed
11	rs4372482	117839307	C	0.437	*IL10RA* (20 kb upstream)	0.5644	0.1092	2.76E-07	Imputed
11	rs4938466	117845068	G	0.432	*IL10RA* (20 kb upstream)	0.5613	0.1104	4.27E-07	Imputed
1	rs35118879	150600680	C	0.931	*ENSA* (intron)	1.0880	0.2159	5.32E-07	Imputed
1	rs35392872	150547938	G	0.931	*MCL1* (3’-utr)	1.0925	0.2168	5.34E-07	Imputed
1	rs71622702	150514747	C	0.909	*ADAMTSL4* (7 kb upstream)	0.9490	0.1902	6.83E-07	Imputed
1	rs11803990	150539706	C	0.924	*ADAMTSL4* (7 kb downstream)	1.0237	0.2076	9.19E-07	Imputed

^a^F: frequency of the effect allele

**Table 4:** List of the most significant associations with IS (*P*-value < E-06) in the AGRE cohort

CHR	SNP	BP (hg19)	Effect allele	F^a^	Nearest Gene	beta	se	P(wald)	type
8	rs62503729	27083792	A	0.901	*STMN4* (10 kb downstream)	-1.0192	0.1925	1.39E-07	Imputed
8	rs13278976	27085479	T	0.901	*STMN4* (10 kb downstream)	-1.0129	0.1924	1.64E-07	Imputed
8	rs13270725	27085587	C	0.901	*STMN4* (10 kb downstream)	-1.0129	0.1924	1.64E-07	Imputed
8	rs35240189	27079834	G	0.896	*STMN4* (10 kb downstream)	-0.9852	0.1872	1.65E-07	Imputed
8	rs2322600	27139408	G	0.14	*TRIM35* (3 kb downstream)	0.9316	0.1772	1.71E-07	Imputed
8	rs34759235	27071936	G	0.896	*STMN4* (10 kb downstream)	-0.9748	0.1879	2.45E-07	Imputed
8	rs4733041	27113678	A	0.894	*STMN4* (intron)	-0.9802	0.1897	2.75E-07	Imputed
8	rs11135988	27112923	T	0.894	*STMN4* (intron)	-0.9798	0.1897	2.78E-07	Imputed
8	rs12542830	27111998	C	0.894	*STMN4* (intron)	-0.9792	0.1897	2.81E-07	Imputed
8	rs12542220	27111546	C	0.894	*STMN4* (intron)	-0.9789	0.1897	2.82E-07	Imputed
8	rs12542148	27111265	C	0.894	*STMN4* (intron)	-0.9787	0.1896	2.83E-07	Imputed
8	rs12675791	27094644	G	0.894	*STMN4* (intron)	-0.9755	0.1892	2.89E-07	Imputed
8	rs12549968	27108413	C	0.894	*STMN4* (intron)	-0.9767	0.1895	2.94E-07	Imputed
8	rs2322606	27186923	A	0.169	*PTK2B* (intron)	0.8255	0.1602	2.97E-07	Genotyped^a^
5	rs747919	66792177	C	0.426	*LOC359819* (200 kb)	0.6232	0.1210	2.98E-07	Imputed
8	rs12550034	27096145	G	0.894	*STMN4* (intron)	-0.9740	0.1891	2.99E-07	Imputed
6	rs11970233	7962158	C	0.802	*TXNDC5* (intron)	-0.7461	0.1451	3.11E-07	Imputed
8	rs3739213	27100014	G	0.894	*STMN4* (intron)	-0.9739	0.1894	3.11E-07	Imputed
8	rs12541011	27104497	C	0.894	*STMN4* (intron)	-0.9734	0.1893	3.12E-07	Imputed
8	rs4733039	27101046	C	0.894	*STMN4* (intron)	-0.9725	0.1892	3.15E-07	Imputed
8	rs1562331	27097746	G	0.894	*STMN4* (intron)	-0.9697	0.1889	3.27E-07	Genotyped^a^
6	rs9505329	7960742	G	0.803	*TXNDC5* (intron)	-0.7343	0.1451	4.73E-07	Imputed
8	rs10097861	27188518	G	0.19	*PTK2B* (intron)	0.7654	0.1514	4.86E-07	Genotyped^a^
6	rs13437591	7959662	G	0.803	*TXNDC5* (intron)	-0.7324	0.1451	5.14E-07	Imputed
6	rs155476	7961189	C	0.745	*TXNDC5* (intron)	-0.6684	0.1327	5.36E-07	Genotyped^a^
6	rs429530	7956571	G	0.803	*TXNDC5* (intron)	-0.7304	0.1453	5.63E-07	Imputed
8	rs11782061	27188980	T	0.191	*PTK2B* (intron)	0.7618	0.1516	5.66E-07	Imputed
6	rs155495	7945804	G	0.805	*TXNDC5* (intron)	-0.7299	0.1458	6.30E-07	Imputed
6	rs155500	7949075	C	0.805	*TXNDC5* (intron)	-0.7294	0.1461	6.76E-07	Imputed
8	rs4733043	27113883	G	0.888	*STMN4* (intron)	-0.9248	0.1864	7.95E-07	Imputed
8	rs12541668	27105399	C	0.901	*STMN4* (intron)	-0.9727	0.1967	8.63E-07	Imputed
8	rs3739214	27101279	C	0.901	*STMN4* (intron)	-0.9715	0.1966	8.73E-07	Imputed
6	rs155491	7943686	G	0.804	*TXNDC5* (intron)	-0.7166	0.1452	8.95E-07	Imputed

^a^F: frequency of the effect allele

**Fig. 4:**
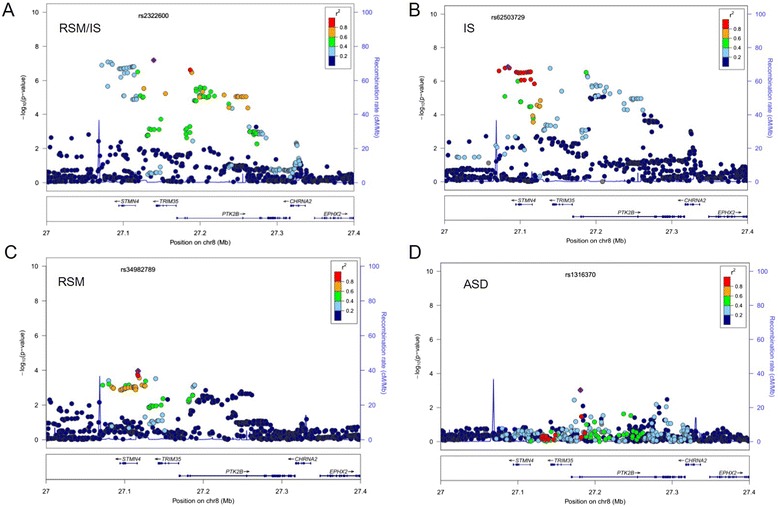
Regional plots showing association mapping results for SNPs located on chromosome 8p21.1-8p21.2 region for RSM/IS (**a**), IS (**b**), RSM (**c**) and ASD (**d**). *Each filled circle represents the P-value for one SNP, with the top SNP, represented by a purple diamond and additional associated SNPs represented by colors showing their degree of linkage disequilibrium (r^2^) with the top SNP (as estimated internally by the Locus Zoom program based on data from CEU (Utah residents of Northern and Western European ancestry HapMap haplotypes) population. Genes within the region are shown in the lower panel, and the unbroken blue line indicates the recombination rate within the region. *Association with common variants in this region and ASD diagnosis were analyzed according to previously reported association analyses with the AGRE pedigrees [9] using Pedigree Disequilibrium Test (PDT) [83]

For RSM/IS, the top-ranked SNPs were identified within 8p21.2-8p21.1, completely overlapped with the most highly associated region for IS (Fig. 3c), Table 5). As shown in regional plots of association signals (Fig. 4a b c), association with 8p21.2-8p21.1 region SNPs is greater for RSM/IS than for IS or RSM, suggesting that the multivariate model including RSM and IS might provide greater power for detecting associations. Since no significant family genetic effect was detected for RSM in our analysis, association in the multivariate association analysis was likely driven by the IS category. It should also be noted that this region did not show any significant association with ASD diagnosis after we re-analyzed the association for this region in AGRE cohort following the earlier analysis [9] (Fig. 4d). Based on these observations, we focused subsequence analyses on the 8p21.2-8p21.1 region and hypothesized that common variants in this region may be novel candidate loci for RRB, especially IS.

**Table 5:** List of the most significant associations with RSM/IS (*P*-value < 5.0E-07) in the AGRE cohort

CHR	SNP	BP (hg19)	Effect allele	F^a^	Nearest Gene	P(wald)	Type
8	rs2322600	27139408	G	0.14	*TRIM35* (3 kb downstream)	6.48E-08	Imputed
8	rs35240189	27079834	G	0.104	*STMN4* (10 kb downstream)	8.18E-08	Imputed
8	rs62503729	27083792	A	0.099	*STMN4* (10 kb downstream)	8.83E-08	Imputed
8	rs13278976	27085479	T	0.099	*STMN4* (10 kb downstream)	1.12E-07	Imputed
8	rs13270725	27085587	C	0.099	*STMN4* (10 kb downstream)	1.13E-07	Imputed
8	rs34759235	27071936	G	0.104	*STMN4* (10 kb downstream)	1.27E-07	Imputed
8	rs4733041	27113678	A	0.106	*STMN4* (intron)	1.53E-07	Imputed
8	rs11135988	27112923	T	0.106	*STMN4* (intron)	1.55E-07	Imputed
8	rs12542830	27111998	C	0.106	*STMN4* (intron)	1.57E-07	Imputed
8	rs12542220	27111546	C	0.106	*STMN4* (intron)	1.58E-07	Imputed
8	rs12542148	27111265	C	0.106	*STMN4*(intron)	1.59E-07	Imputed
8	rs12549968	27108413	C	0.106	*STMN4* (intron)	1.69E-07	Imputed
8	rs12541011	27104497	C	0.106	*STMN4* (intron)	1.83E-07	Imputed
8	rs4733039	27101046	C	0.106	*STMN4* (intron)	1.90E-07	Imputed
8	rs3739213	27100014	G	0.106	*STMN4* (intron)	1.96E-07	Imputed
8	rs12550034	27096145	G	0.106	*STMN4* (intron)	2.06E-07	Imputed
8	rs1562331	27097746	G	0.106	*STMN4* (intron)	2.06E-07	Genotyped^a^
8	rs12675791	27094644	G	0.106	*STMN4* (intron)	2.06E-07	Imputed
8	rs2322606	27186923	A	0.169	*PTK2B* (intron)	2.40E-07	Genotyped^a^
8	rs12546017	27118605	A	0.105	*STMN4* (3 kb downstream)	3.12E-07	Imputed
8	rs10097861	27188518	G	0.19	*PTK2B* (intron)	3.20E-07	Genotyped^a^
8	rs11782061	27188980	T	0.191	*PTK2B* (intron)	3.46E-07	Imputed
8	rs12541668	27105399	C	0.099	*STMN4* (intron)	3.54E-07	Imputed
8	rs3739214	27101279	C	0.099	*STMN4* (5’-utr)	3.72E-07	Imputed
11	rs11512467	117835174	C	0.423	*IL10RA* (20 kb upstream)	3.77E-07	Imputed
6	rs11970233	7962158	C	0.198	*TXNDC5* (intron)	4.10E-07	Imputed
11	rs12790242	117842292	G	0.421	*IL10RA* (20 kb upstream)	4.16E-07	Imputed
8	rs4733043	27113883	G	0.112	*STMN4* (intron)	4.75E-07	Imputed

^a^F: frequency of the effect allele

#### Replication (SSC) dataset

Based on PCA of 11 ADI-R scores in the SSC dataset, we identified the same two RRB subcategories, RSM and IS, observed in the AGRE dataset (Additional file 6). However, no statistically significant signals were detected for association with RSM、IS or RSM/IS in GEMMA univariate or multivariate analysis of 8p21.2-8p21.1 region SNPs. The top-ranked SNP rs17057065 for RSM/IS with *P*-value 0.041 is located in an intron of *PTK2B* (Regional plot showing association *P*-values for 8p21.2-8p21.1 region SNPs is shown in Additional file 7). The lack of association in the SSC dataset may be caused by between-study heterogeneity. SSC probands included in present analysis are from simplex families, in contrast to 90 % of the probands in the AGRE cohort who are from multiplex families. Thus, the relatedness might increase the power for association analysis in AGRE sample. An additional source of heterogeneity comes from the age distributions of the probands in these two dataset, for which the sample means are significantly different (t = 34.3, *P-*value < 0.001).

Although the associations we reported for 8p21.2-8p21.1 were not observed in SSC dataset, previous observations provide biological plausibility for the contribution of chromosome 8p and 8p21.2-p21.1 to ASD. Chromosome 8p is known to harbor numerous genes implicated in developmental neuropsychiatric disorders, including schizophrenia and ASD [53]. In the largest schizophrenia linkage analysis to date, the 8p21.2-p21.1 region was found to be associated with schizophrenia (*P*-value = 4.00E-04) [54]. In a subsequent publication, the same group reported significant associations for SNPs in and around *DPYSL2* and *ADRA1A*, 8p21.2-p21.1 region genes previously associated with schizophrenia in family-based and case-control association studies [55, 56]; the strongest associated SNP (rs7817434; *P*-value = 3.01E-04) is located 377 kb from the top signal in our analysis (rs2322600) [57]. Recent meta-analyses of CNV and GWA studies results suggest that there are both clinical and biological links between autism and schizophrenia [58, 59], so it is highly plausible that common variants in this region contribute to both ASD and schizophrenia. Significantly, a subset of schizophrenia patients display multiple repetitive behaviors [60].

#### Genomic features of the 8p21.2-8p21.1 region

The SNP showing the most significant association for RSM/IS, rs2322600, has a P-value just shy of genome-wide significance (*P*-value = 6.48E-08). In addition, twenty-four SNPs in the same region have *P*-values for association smaller than 5E − 07, including the top associated SNP for IS (rs62503729) and three genotyped SNPs (rs1562331, rs2322606 and rs10097861) that are in linkage disequilibrium with the top three SNPs (Additional file 8). Together, these 25 SNPs span a 117 kb region that contains three genes: *STMN4*, *TRIM35* and *PTK2B. STMN4* (stathmin-like 4) interacts directly with microtubules, causing a switch from a straight to a curved conformation that has been proposed to promote rapid microtubule depolymerization. According to the BrainCloud study database (http://braincloud.jhmi.edu/), *STMN4* mRNA is highly expressed in both fetal and adult dorsolateral prefrontal cortex (DLPFC) (Additional file 9) [48], consistent with a role in early neuron development [61, 62]. *PTK2B* (Protein Tyrosine Kinase 2 Beta) encodes a major focal adhesion kinase that plays a key role in neuritogenesis and neurite elongation [63]. Because all the top SNPs are located in non-coding gene regions, we hypothesized that these SNPs are linked to genetic variants that regulate the expression *STMN4* or *PTK2B*.

#### Expression quantitative trait locus (eQTL) analyses using adult CEU prefrontal cortex

With the aim of exploring the molecular basis of the observed associations with RSM/IS, we investigated whether our top SNPs or their proxies (r^2^ > 0.7) associate with gene expression in the dorsolateral prefrontal cortex (Brodmann area 46) using expression and genotype data of 112 healthy adult CEU individuals. Top SNPs and multiple proxy SNPs in or near *STMN4* showed nominally significant association between genotype and *STMN4* mRNA expression (rs2322600, *P*-value = 0.023; rs35240189, *P*-value = 0.014; rs62503729, *P*-value = 0.024), with the strongest association represented by rs10097861 (a proxy of rs2322600, r^2^ = 0.76) at a P-value of 0.002. None of the SNPs were associated with the expression of *PTK2B* mRNA. Since expression data in BrainCloud dataset were from adult human brain, it is possible that the genetic variants regulate *PTK2B* mRNA expression only in the developing brain.

#### Bioinformatic evaluation

We queried the RegulomeDB database [64] to assess whether any of the 28 SNPs that associated with the RSM/IS sub-phenotypes (*P*-value < 5E-07, *n* = 28 SNPs) are located in known or predicted regulatory elements, including regions of DNase I hypersensitivity, binding sites for transcription factors and promoter regions that regulate transcription. Two SNPs, rs2322606 and rs10097861, received RegulomeDB likelihood scores of 1f (i.e., mapping to a predicted TF binding site and/or within a DNase I sensitivity peak and correlating with gene expression). Both of these variants, which are located within an intron of *PTK2B*, associated with expression of *BNIP3L* mRNA in a lymphoblastoid cell line and located within DNase I hypersensitivity peaks.

#### Functional enrichment pathway analysis

In total, 252 genes linked to SNPs with nominal associations (*P* < 0.01) with RSM/IS in the multivariate analysis of the AGRE cohort were selected for enrichment analysis using DAVID [see Additional file 10]. Using the DAVID Functional Annotation Clustering tool, we identified 7 annotation term clusters with enrichment scores > 1.3 (equivalent to a nominal *P*-value ≤ 0.05), including two clusters containing pathways crucial for brain development and function [see Additional file 11]. Cluster 1(enrichment score: 1.93) contained several pathways previously implicated in the pathogenesis of ASD, including neuron development (GO:0048666, *P*-value = 0.002797) [65], neuron projection development (GO:0031175, *P*-value = 0.003104) [66] and axon guidance (GO:0007411, *P*-value =0.016). Cluster 3 (enrichment score: 1.75) contained several cell-adhesion pathways, which have also been implicated in the pathogenesis of Autism Spectrum Disorders [9, 67].

As a negative control, we also carried out DAVID-based enrichment analyses using candidate gene lists derived from association analyses of 10 sets of permuted RSM/IS phenotypes (specifically, the phenotype-permuted replicates with the ten highest lambdaGC values). Among 53 annotation term clusters with enrichment scores greater than 1.3 that were obtained for the ten gene sets, only one was related to the brain/neuron development (Additional file 12). These results suggest that the gene list derived from the original, non-permutated association is enriched in brain and plausible ASD-related pathways. Because no enriched annotation term cluster identified in present study survived correction for multiple testing, however, the possibility of false-positive enrichments cannot be excluded.

## Discussion

In this work, analysis of ADR-R RRB item scores in the AGRE and SSC datasets confirmed the existence of two previously identified subcategories in the RRB domain, RSM and IS. GWAS of RSM and IS subcategory scores based on univariate and multivariate mixed models identified common variants within 8p21.2-8p21 as possible susceptibility locus for RRB in the AGRE dataset, but not in the SSC dataset.

Univariate association analysis identified different association patterns for IS and RSM, including signals at 1q21-1q21.2、6p24.3、11q23 and 8p21.2-8p21.1 that have not previously been reported for association with RRB. Several regions in 15q have previously been linked to RSM or IS in linkage analysis, including 15q21.2-q22.2, 15q13.1-q14 [25] and 15q11-q13 [31]. Our analysis failed to detect suggestive associations for RSM or IS at these loci, although a suggestive association with IS was detected for the 15q24.1 region SNP rs138618349 (*P* = 1.99E-06). Lack of replication may reflect difference in populations and/or methodology, i.e., linkage vs. association analysis.

Common variants within chromosome 8p21.2-8p21.1, a locus previously linked to schizophrenia, approached genome-wide significance for RSM/IS and were also the top signals for IS in univariate association analysis. Since many genetic variants linked to ASD have a high degree of pleiotropy (i.e., where one gene affects more than one phenotype), it is reasonable that some genetic variants contribute to both RSM and IS and were detected with higher association magnitude using multivariate association model [68]. Although the associations we reported for 8p21.2-8p21.1 were not observed in the SSC dataset, we should mention that a partial trisomy of 8p(21-23) has been identified in a 6-year-old female with autism [69]. This region is also included in a large (6.14 Mbp) chromosome duplication identified in a patient with autism and self-mutilation [70]. This patient presented with abnormal behaviors, including ritualistic behaviors, self-injury and temper tantrums, consistent with the hypothesis that this chromosomal region contains a dosage-sensitive gene that contributes to RRB phenotypes.

Our top SNPs and multiple proxy SNPs in or near *STMN4* were identified as eQTLs for *STMN4* in human prefrontal cortex, and it is plausible that *STMN4* influences RRB domain phenotypes by modulating neuron development and dendritic microtubule dynamics [71].

Furthermore, based on mRNA expression data from two public databases [64, 72], our top SNP, rs2322600, and several proxy SNPs correlate with expression of *BNIPL3*, a gene located almost 890 kilo base pairs upstream (rs2322600: *P*-value = 9.17E-06). Since long-range regulation of mRNA expression by genetic elements located as far away as 1 Mbp, has been previously described [73, 74], in principle, this gene may also be considered a candidate for RRB. Although *BNIPL3* has not previously been reported to be associated with ASD, it encodes a mitochondrial outer membrane protein that is required for mitochondrial clearance and has been proposed to play a role in hypoxia-induced autophagy [75]. Recent research [67] has shown that children and adolescents with autism have high dendritic spine density in the brain and this excess is due to a defect in dendritic spine “pruning,” a process essential for normal brain development [76]. The same study also showed that the abnormal spine pruning is caused by a defect in autophagy in neurons [67]. Mitochondria localize in both pre- and postsynaptic department (axon terminals and dendritic spines), and mitophagy is crucial for brain development and dendritic spine pruning [77].

*PTK2B* has been widely studied since it was identified as a novel Alzheimer’s disease (AD) candidate gene in a large meta-analysis of AD GWAS [78]. *PTK2B* kinase, a major focal adhesion kinase, regulates the integrity of focal adhesions, which are major compartments for integrating signals for cell growth, apoptosis, and neuron migration, cellular functions essential for normal brain development [79]. Since several neuronal cell-adhesion genes have been identified in rare ASD cases [80, 81] and a GWA study has shown that neuronal cell-adhesion molecules may be collectively associated with ASDs [9], it is plausible that *PTK2B* contributes to ASD through its roles in regulating integrity of focal adhesions.

Because the terms in each enriched cluster identified by the pathway analysis did not survive multiple testing correction (*P*-value > 0.05 after controlling for the false discovery rate (FDR) using the Benjamini-Hochberg method), we could not identify specific biological pathways that contribute to the development of RRB in ASD. However, DAVID analysis included *PTK2B* among the top three enriched terms in Cluster1(Additional file 11), based on its function in focal adhesion formation and regulation of adherens junction dynamics by phosphorylation switches [82], providing evidence that *PTK2B* is a plausible candidate gene for RRB.

## Conclusions

In this study, univariate and multivariate genome-wide association studies of RRB subcategories using data from an AGRE ASD cohort enabled the detection of associated SNPs at 8p21.2-8p21.1. This region contained 25 genotyped or imputed SNPs with *P*-values for association with RSM/IS < 5E-07, with the top SNP (*P*-value = 6.48E-08) just missing genome-wide significance. Notably, 8p21.2-8p21 has previously been linked to schizophrenia and our top SNPs are located in close proximity and/or correlate with the expression of several genes with plausible connections to ASD and RRB. Because association signals in this chromosome region were not detected in the SSC ASD dataset, however, more work will be required to validate the possible contributions of common variants in 8p21.2-8p21.1 to RRB or ASD.

## Additional files

Additional file 1:
**Two-dimensional Multidimensional Scaling (MDS) plot of the AGRE population.** (DOCX 88 kb) Additional file 2:
**Sample information for 1,335 individuals in the AGRE cohort.** (XLSX 42 kb) Additional file 3:
**Sample information for 2,588 individuals in SSC.** (XLSX 41 kb) Additional file 4:
**Current scores for eleven ADI-R items in AGRE that assess restricted repetitive behaviors.** (XLSX 68 kb) Additional file 5:
**Score distributions of the RSM and IS subcategories in the AGRE cohort.** (DOCX 60 kb) Additional file 6:
**Two-Factor solution for the Restricted and Repetitive behaviors in SSC using PCA with varimax rotation.** (DOCX 15 kb) Additional file 7:
**Regional plot showing association mapping results for association with RSM/IS for SNPs located within chromosome 8p21.1-8p21.2 in the SSC dataset.** (DOCX 67 kb) Additional file 8:
**Haploview linkage disequilibrium (LD) plots for genotyped SNPs and the top three associated SNPs in** **Table** 5 **of the manuscript.** (DOCX 41 kb) Additional file 9:
**Developmental time course of** ***STMN4*** **mRNA expression in adult CEU (Caucasian and European descent) human prefrontal cortex.** (DOCX 26 kb) Additional file 10:
**Candidate gene list for Functional Enrichment Analysis.** (XLSX 21 kb) Additional file 11:
**Enrichment of functional annotation terms identified using DAVID software for genes linked to SNPs that nominally associate with RSM/IS.** (DOCX 27 kb) Additional file 12:
**Enrichment of functional annotation terms identified using DAVID software for genes linked to SNPs from ten results of phenotype-permuted datasets.** (XLSX 53 kb)

## Abbreviations

ADI-R: Autism Diagnostic Interview-Revised
ADOS: Autism Diagnostic Observation Schedule
AGRE: Autism Genetic Resource Exchange
ASD: Autism Spectrum Disorders
DAVID: Database for Annotation, Visualization and Integrated Discovery
ES: enrichment score
eQTL: expression quantitative trait loci
GEMMA: Genome-wide Efficient Mixed-Model Association
IS: insistence on sameness
ICCs: intraclass correlations
lambdaGC: Lambda genomic conttol
MDS: multidimensional scaling
PCA: Principal Components Analysis
RSM: Repetitive Sensory Motor
RRB: restricted and repetitive behaviors
SSC: Simons Simplex Collection
