# Supplementary material for: Evidence for contribution of common genetic variants within chromosome 8p21.2-8p21.1 to restricted and repetitive behaviors in autism spectrum disorders
Source: BMC Genomics. 2016 Mar 1;17:163. doi: 10.1186/s12864-016-2475-y (PMC4774106; doi:10.1186/s12864-016-2475-y)
Supplement: Additional file 1: — Two-dimensional Multidimensional Scaling (MDS) plot of the AGRE population. (DOCX 88 kb) [file 12864_2016_2475_MOESM1_ESM.docx]

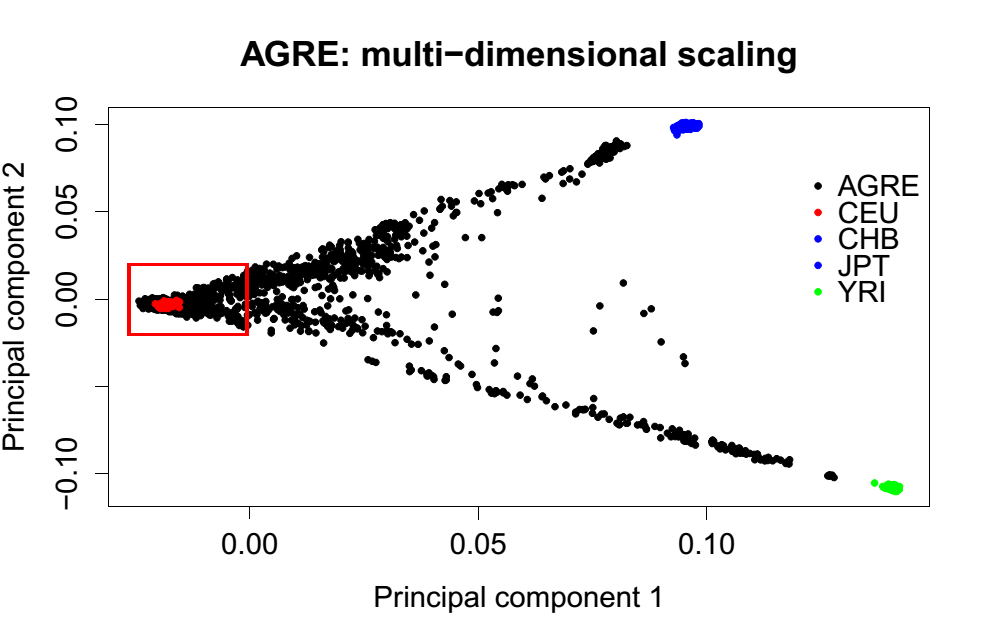


Additional file 1 **-** Two-dimensional Multidimensional Scaling (MDS) plot of the AGRE population. Black dots represent AGRE individuals; colored dots represent HAPMAP3 project populations. CEU: Utah residents with Northern and Western European ancestry from the CEPH collection; CHB: Han Chinese in Beijing; JPT: Japanese in Tokyo; YRI: Yoruba in Ibadan, Nigeria.
