# Supplementary material for: Evidence for contribution of common genetic variants within chromosome 8p21.2-8p21.1 to restricted and repetitive behaviors in autism spectrum disorders
Source: BMC Genomics. 2016 Mar 1;17:163. doi: 10.1186/s12864-016-2475-y (PMC4774106; doi:10.1186/s12864-016-2475-y)
Supplement: Additional file 5: — Score distributions of the RSM and IS subcategories in the AGRE cohort. (DOCX 60 kb) [file 12864_2016_2475_MOESM5_ESM.docx]

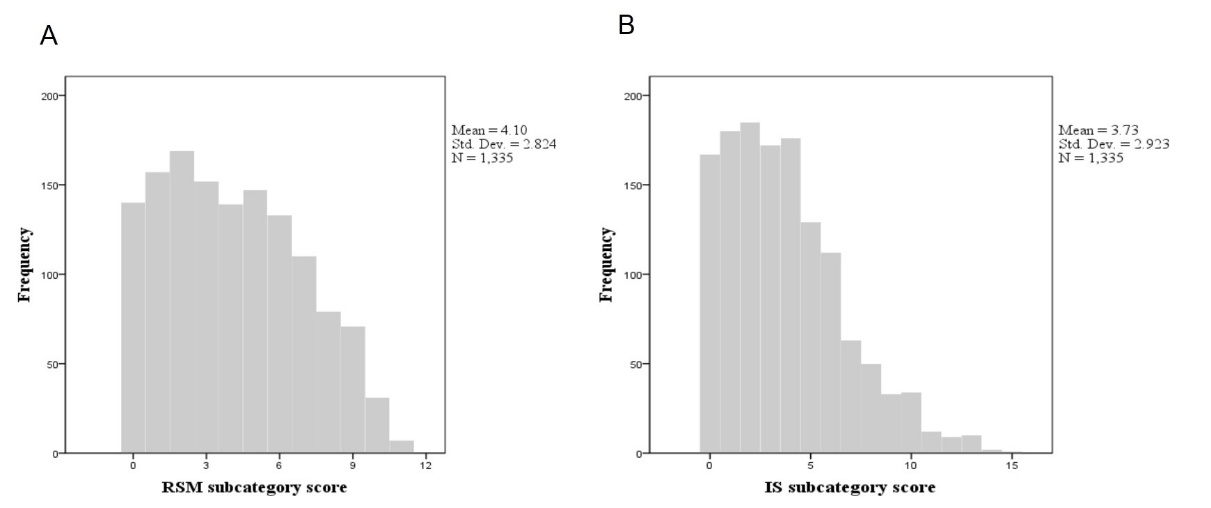


Additional file 5 **–** Distribution of RSM subcategory scores (left) and IS subcategory scores (right) in the AGRE sample
