# Supplementary material for: Evidence for contribution of common genetic variants within chromosome 8p21.2-8p21.1 to restricted and repetitive behaviors in autism spectrum disorders
Source: BMC Genomics. 2016 Mar 1;17:163. doi: 10.1186/s12864-016-2475-y (PMC4774106; doi:10.1186/s12864-016-2475-y)
Supplement: Additional file 6: — Two-Factor solution for the Restricted and Repetitive behaviors in SSC using PCA with varimax rotation. (DOCX 15 kb) [file 12864_2016_2475_MOESM6_ESM.docx]

Additional file 6. Two-Factor solution for the Restricted and Repetitive Behaviors in SSC using PCA with varimax rotation*

| ADI-R items from RRB domain | Factor 1  Repetitive Sensory Motor (RSM) | Factor 2  Insistence On Sameness (IS) |
| --- | --- | --- |
| Unusual sensory interests | **0.57** | 0.18 |
| Repetitive use of objects | **0.54** | 0.13 |
| Hand and finger mannerisms | **0.46** | 0.02 |
| Complex mannerisms or stereotyped body movements | **0.43** | 0.09 |
| Difficulties with change | 0.08 | **0.65** |
| Resistance to change | 0.05 | **0.39** |
| Circumscribed interests | -0.03 | **0.30** |
| Compulsions/rituals | 0.20 | **0.41** |
| Abnormal/Idiosyncratic response | 0.21 | **0.47** |
| Unusual preoccupations | 0.07 | 0.29 |
| Sensitivity to noise | 0.16 | **0.33** |

Note: Factor loadings of those items that exceed 0.30 are bolded.

*Chi Square [df = 34] = 113.62, P-value = 1.65e-10
