# Supplementary material for: Evidence for contribution of common genetic variants within chromosome 8p21.2-8p21.1 to restricted and repetitive behaviors in autism spectrum disorders
Source: BMC Genomics. 2016 Mar 1;17:163. doi: 10.1186/s12864-016-2475-y (PMC4774106; doi:10.1186/s12864-016-2475-y)
Supplement: Additional file 7: — Regional plot showing association mapping results for association with RSM/IS for SNPs located within chromosome 8p21.1-8p21.2 in the SSC dataset. (DOCX 67 kb) [file 12864_2016_2475_MOESM7_ESM.docx]

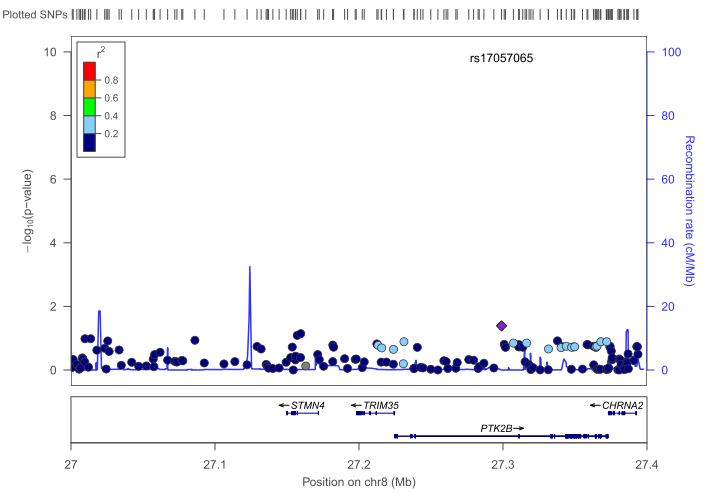


Additional file 7. Regional plot showing association mapping results for SNPs located within chromosome 8p21.1-8p21.2 region for RSM/IS in SSC dataset. Each filled circle represents the P-value for one SNP, with the top SNP, represented by a purple diamond and additional associated SNPs represented by colors showing their degree of linkage disequilibrium (r^2^) with the top SNP (as estimated internally by the Locus Zoom program based on data from CEU (Utah residents of Northern and Western European ancestry HapMap haplotypes) population. Genes within the region are shown in the lower panel, and the unbroken blue line indicates the recombination rate within the region.
