# Supplementary material for: Evidence for contribution of common genetic variants within chromosome 8p21.2-8p21.1 to restricted and repetitive behaviors in autism spectrum disorders
Source: BMC Genomics. 2016 Mar 1;17:163. doi: 10.1186/s12864-016-2475-y (PMC4774106; doi:10.1186/s12864-016-2475-y)
Supplement: Additional file 8: — Haploview linkage disequilibrium (LD) plots for genotyped SNPs and the top three associated SNPs in Table 5 of the manuscript. (DOCX 41 kb) [file 12864_2016_2475_MOESM8_ESM.docx]

.


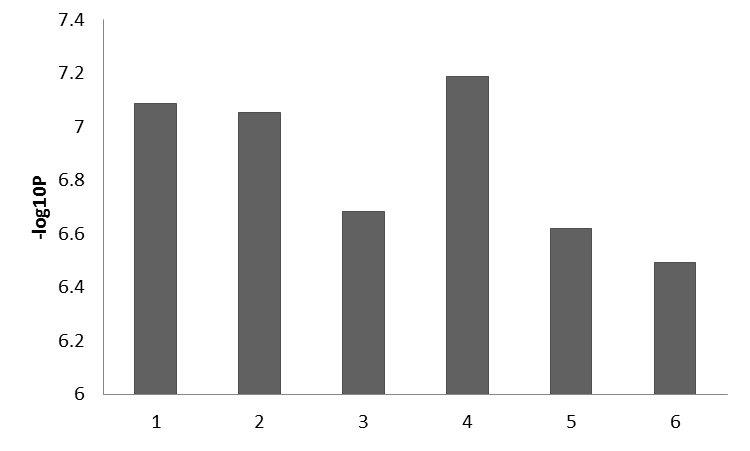


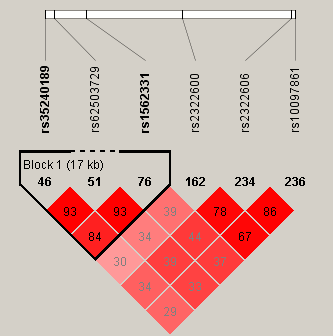


Additional file 8. Haploview linkage disequilibrium (LD) plots for genotyped SNPs and the top three associated SNPs in Table 5 of the manuscript. (top) Bar plot shows the –log10 transformed GEMMA P-values of the genotyped SNPs and the top three SNPs in Table 5. (bottom) LD block comprising the 6 SNPs. Each box contains the estimated value of the pairwise r^2^ LD constant, with the intensity of the red color representing stronger LD. : Genotyped SNPs.
