# Supplementary material for: Evidence for contribution of common genetic variants within chromosome 8p21.2-8p21.1 to restricted and repetitive behaviors in autism spectrum disorders
Source: BMC Genomics. 2016 Mar 1;17:163. doi: 10.1186/s12864-016-2475-y (PMC4774106; doi:10.1186/s12864-016-2475-y)
Supplement: Additional file 9: — Developmental time course of STMN4 mRNA expression in adult CEU (Caucasian and European descent) human prefrontal cortex. (DOCX 26 kb) [file 12864_2016_2475_MOESM9_ESM.docx]

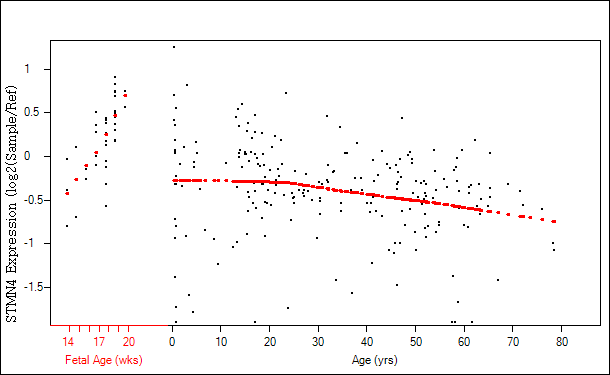


Additional file 9 **-** Developmental time course of *STMN4* mRNA expression in adult CEU (Caucasian and European descent) human prefrontal cortex. The x-axis represents the time course, with fetal ages in weeks and postnatal ages in years and the y-axis represents adjusted expression values for *STMN4* mRNA, with the effects of potentially confounding variables eliminated from the normalized expression data for *STMN4* using surrogate variable analysis. Each black dot represents an individual subject. The red dots represent Loess fit (separate fit for fetal data and for postnatal data). The plots were downloaded from the BrainCloud website (http://braincloud.jhmi.edu/)[[1](#_ENREF_1)]. N = 268, including 38 fetal samples.

1. Numata S, Ye T, Hyde TM, Guitart-Navarro X, Tao R, Wininger M, Colantuoni C, Weinberger DR, Kleinman JE, Lipska BK: **DNA methylation signatures in development and aging of the human prefrontal cortex**. *American journal of human genetics* 2012, **90**(2):260-272.
