# Supplementary material for: Evidence for contribution of common genetic variants within chromosome 8p21.2-8p21.1 to restricted and repetitive behaviors in autism spectrum disorders
Source: BMC Genomics. 2016 Mar 1;17:163. doi: 10.1186/s12864-016-2475-y (PMC4774106; doi:10.1186/s12864-016-2475-y)
Supplement: Additional file 11: — Enrichment of functional annotation terms identified using DAVID software for genes linked to SNPs that nominally associate with RSM/IS. (DOCX 27 kb) [file 12864_2016_2475_MOESM11_ESM.docx]

Additional file 11. Enrichment of functional annotation terms identified using DAVID software [[1](#_ENREF_1), [2](#_ENREF_2)] for genes linked to SNPs that nominally associate with RSM/IS. (Enrichment threshold: ES >1.3)

| **Cluster 1** | **Enrichment Score: 1.93** |  |  |  |
| --- | --- | --- | --- | --- |
| Category | Term | Count | P-value | Benjamini |
| GOTERM_BP_FAT | GO:0030030~cell projection organization | 15 (PTK2B included) | 6.33E-04 | 0.639723 |
| GOTERM_BP_FAT | GO:0048666~neuron development | 13(PTK2B included) | 0.002797 | 0.895403 |
| GOTERM_BP_FAT | GO:0031175~neuron projection development | 11(PTK2B included) | 0.003104 | 0.811874 |
| GOTERM_BP_FAT | GO:0032989~cellular component morphogenesis | 14 | 0.003685 | 0.774123 |
| GOTERM_BP_FAT | GO:0000902~cell morphogenesis | 13 | 0.004145 | 0.737937 |
| GOTERM_BP_FAT | GO:0006928~cell motion | 15 | 0.00658 | 0.830279 |
| GOTERM_BP_FAT | GO:0048858~cell projection morphogenesis | 10 | 0.007284 | 0.814267 |
| GOTERM_BP_FAT | GO:0030182~neuron differentiation | 14 | 0.008241 | 0.772864 |
| GOTERM_BP_FAT | GO:0048667~cell morphogenesis involved in neuron differentiation | 9 | 0.008858 | 0.761728 |
| GOTERM_BP_FAT | GO:0032990~cell part morphogenesis | 10 | 0.009601 | 0.756764 |
| GOTERM_BP_FAT | GO:0048812~neuron projection morphogenesis | 9 | 0.009864 | 0.735961 |
| GOTERM_BP_FAT | GO:0007411~axon guidance | 6 | 0.016722 | 0.743131 |
| GOTERM_BP_FAT | GO:0007409~axonogenesis | 8 | 0.018302 | 0.757784 |
| GOTERM_CC_FAT | GO:0030424~axon | 7 | 0.019146 | 0.480486 |
| GOTERM_BP_FAT | GO:0000904~cell morphogenesis involved in differentiation | 9 | 0.020549 | 0.766657 |
| GOTERM_CC_FAT | GO:0043005~neuron projection | 10 | 0.038014 | 0.650155 |
| GOTERM_CC_FAT | GO:0042995~cell projection | 16 | 0.042974 | 0.661136 |
| KEGG_PATHWAY | hsa04360:Axon guidance | 5 | 0.126272 | 0.972044 |
| GOTERM_BP_FAT | GO:0007626~locomotory behavior | 6 | 0.335476 | 0.987259 |
| **Cluster 2** | **Enrichment Score: 1.86** |  |  |  |
| Category | Term | Count | P-value | Benjamini |
| GOTERM_MF_FAT | GO:0005044~scavenger receptor activity | 5 | 0.003853 | 0.814175 |
| UP_SEQ_FEATURE | domain:SRCR | 3 | 0.013664 | 0.987354 |
| INTERPRO | IPR001190:Speract/scavenger receptor | 3 | 0.051099 | 0.984253 |
| **Cluster 3** | **Enrichment Score: 1.75** |  |  |  |
| Category | Term | Count | P-value | Benjamini |
| GOTERM_CC_FAT | GO:0005912~adherens junction | 8 | 0.004518 | 0.706884 |
| GOTERM_CC_FAT | GO:0016323~basolateral plasma membrane | 9 | 0.005578 | 0.396678 |
| GOTERM_CC_FAT | GO:0070161~anchoring junction | 8 | 0.007885 | 0.415111 |
| GOTERM_CC_FAT | GO:0005925~focal adhesion | 6 | 0.011331 | 0.460791 |
| GOTERM_CC_FAT | GO:0005924~cell-substrate adherens junction | 6 | 0.013222 | 0.451833 |
| GOTERM_CC_FAT | GO:0030055~cell-substrate junction | 6 | 0.016441 | 0.473645 |
| GOTERM_CC_FAT | GO:0030054~cell junction | 12 | 0.082433 | 0.810863 |
| SP_PIR_KEYWORDS | cell junction | 8 | 0.254705 | 0.917177 |
| **Cluster 4** | **Enrichment Score: 1.74** |  |  |  |
| Category | Term | Count | P-value | Benjamini |
| GOTERM_BP_FAT | GO:0030030~cell projection organization | 15 | 6.33E-04 | 0.639723 |
| GOTERM_BP_FAT | GO:0006928~cell motion | 15 | 0.00658 | 0.830279 |
| GOTERM_BP_FAT | GO:0016477~cell migration | 10 | 0.015101 | 0.744033 |
| GOTERM_BP_FAT | GO:0051674~localization of cell | 10 | 0.02781 | 0.837752 |
| GOTERM_BP_FAT | GO:0048870~cell motility | 10 | 0.02781 | 0.837752 |
| GOTERM_BP_FAT | GO:0040012~regulation of locomotion | 7 | 0.051742 | 0.925374 |
| KEGG_PATHWAY | hsa04670:Leukocyte transendothelial migration | 4 | 0.258366 | 0.970411 |
| **Cluster 5** | **Enrichment Score: 1.68** |  |  |  |
| Category | Term | Count | P-value | Benjamini |
| GOTERM_BP_FAT | GO:0009719~response to endogenous stimulus | 13 | 0.011098 | 0.749398 |
| GOTERM_BP_FAT | GO:0010033~response to organic substance | 19 | 0.011625 | 0.692141 |
| GOTERM_BP_FAT | GO:0009725~response to hormone stimulus | 10 | 0.070683 | 0.927631 |
| **Cluster 6** | **Enrichment Score: 1.39** |  |  |  |
| Category | Term | Count | P-value | Benjamini |
| INTERPRO | IPR008957:Fibronectin, type III-like fold | 9 | 0.003588 | 0.863474 |
| UP_SEQ_FEATURE | domain:Fibronectin type-III 2 | 6 | 0.026951 | 0.986956 |
| UP_SEQ_FEATURE | domain:Fibronectin type-III 1 | 6 | 0.027736 | 0.978278 |
| INTERPRO | IPR003961:Fibronectin, type III | 7 | 0.044458 | 0.98499 |
| UP_SEQ_FEATURE | domain:Fibronectin type-III 4 | 4 | 0.046194 | 0.993316 |
| SMART | SM00060:FN3 | 7 | 0.067558 | 0.990113 |
| INTERPRO | IPR003962:Fibronectin, type III subdomain | 3 | 0.076854 | 0.992719 |
| UP_SEQ_FEATURE | domain:Fibronectin type-III 3 | 4 | 0.092537 | 0.996923 |
| UP_SEQ_FEATURE | domain:Fibronectin type-III 5 | 3 | 0.1148 | 0.997794 |
| **Cluster 7** | **Enrichment Score: 1.32** |  |  |  |
| Category | Term | Count | P-value | Benjamini |
| INTERPRO | IPR011510:Sterile alpha motif homology 2 | 4 | 0.017775 | 0.963556 |
| INTERPRO | IPR001660:Sterile alpha motif SAM | 5 | 0.03727 | 0.98513 |
| SMART | SM00454:SAM | 5 | 0.051801 | 0.999107 |
| INTERPRO | IPR013761:Sterile alpha motif-type | 4 | 0.086115 | 0.993186 |
| UP_SEQ_FEATURE | domain: SAM | 4 | 0.087511 | 0.997027 |

Abbreviations: ES, enrichment score; Benjamini, Benjamini multiple testing correction value; GOTERM_BP_FAT (lower levels of biological process ontology), GOTERM_CC_FAT (lower levels of cellular component ontology), GOTERM_MF_FAT (lower levels of molecular function ontology), generated by the DAVID team. This subset is missing the broadest GO terms for ease of data interpretation; DAVID, Database for Annotation, Visualization, and Integrated Discovery; P-value, modified Fisher's exact test;

1. Huang da W, Sherman BT, Lempicki RA: **Systematic and integrative analysis of large gene lists using DAVID bioinformatics resources**. *Nature protocols* 2009, **4**(1):44-57.

2. Huang da W, Sherman BT, Lempicki RA: **Bioinformatics enrichment tools: paths toward the comprehensive functional analysis of large gene lists**. *Nucleic acids research* 2009, **37**(1):1-13.
